# Evidence for impulsivity in the Spontaneously Hypertensive Rat drawn from complementary response-withholding tasks

**DOI:** 10.1186/1744-9081-4-7

**Published:** 2008-02-08

**Authors:** Federico Sanabria, Peter R Killeen

**Affiliations:** 1 Department of Psychology, Arizona State University, PO Box 871104 Tempe, AZ 85287-1104, USA

## Abstract

**Background:**

The inability to inhibit reinforced responses is a defining feature of ADHD associated with impulsivity. The Spontaneously Hypertensive Rat (SHR) has been extolled as an animal model of ADHD, but there is no clear experimental evidence of inhibition deficits in SHR. Attempts to demonstrate these deficits may have suffered from methodological and analytical limitations.

**Methods:**

We provide a rationale for using two complementary response-withholding tasks to doubly dissociate impulsivity from motivational and motor processes. In the lever-holding task (LHT), continual lever depression was required for a minimum interval. Under a differential reinforcement of low rates schedule (DRL), a minimum interval was required between lever presses. Both tasks were studied using SHR and two normotensive control strains, Wistar-Kyoto (WKY) and Long Evans (LE), over an overlapping range of intervals (1 – 5 s for LHT and 5 – 60 s for DRL). Lever-holding and DRL performance was characterized as the output of a mixture of two processes, timing and iterative random responding; we call this account of response inhibition the Temporal Regulation (TR) model. In the context of TR, impulsivity was defined as a bias toward premature termination of the timed intervals.

**Results:**

The TR model provided an accurate description of LHT and DRL performance. On the basis of TR parameter estimates, SHRs were more impulsive than LE rats across tasks and target times. WKY rats produced substantially shorter timed responses in the lever-holding task than in DRL, suggesting a motivational or motor deficit. The precision of timing by SHR, as measured by the variance of their timed intervals, was excellent, flouting expectations from ADHD research.

**Conclusion:**

This research validates the TR model of response inhibition and supports SHR as an animal model of ADHD-related impulsivity. It indicates, however, that SHR's impulse-control deficit is not caused by imprecise timing. The use of ad hoc impulsivity metrics and of WKY as control strain for SHR impulsivity are called into question.

## Background

Attention Deficit Hyperactivity Disorder (ADHD) is characterized by age-inappropriate levels of inattention, impulsivity, and hyperactivity [1]. The most heavily cited theoretical account of ADHD claims that "the essential impairment in ADHD is a deficit involving response inhibition" [2]. Inhibition, according to Barkley's theory, comprises three processes: (a) Inhibiting the *initiation *of responses that have been reinforced in the past but that are currently inappropriate; for example, restraining from playing in classroom; (b) Discontinuing *ongoing *responses that are no longer functional, as when loud talking is halted at the beginning of a lecture; (c) Initiation and maintenance of behaviors that *compete *with the proscribed response, as opening a conversation could distract from the temptation to skip lines, or to respond aggressively. According to this theory [2], deficits in response inhibition yield the executive functioning profile that characterizes ADHD. Most current models of ADHD regularly compare their claims against those of the executive functions model, whether or not they subscribe to it [3-7].

The ability to inhibit reinforced responses is widely studied using the Differential Reinforcement of Low rates (DRL) preparation in both humans [8-13] and animals [14-18]. In DRL, a response (pressing a button or a lever) is reinforced only after a specified interval has elapsed since the last response. Premature responses are often labeled *impulsive*. The opposite of impulsivity, self control, is typically measured as *efficiency*: the proportion of inter-response times (IRT) longer than the target interval. It is important to note that efficiency does not differentiate between IRTs that are just longer than the target interval from those much longer; such very long lapses may result from task delinquency rather than self-control, and, by decreasing the rate of reinforcement, are themselves a source of inefficiency.

The relation between DRL efficiency and ADHD is unclear. Gordon [9] found that 6–8 year old boys rated as hyperactive by their teachers were less efficient in DRL with a 6-s target interval (DRL 6 s) than was a control group. Mancebo [19] found similar results using DRL 5 s. With sufficient training, however, children with ADHD in Mancebo's study acquired behavioral strategies that brought their performance up to the level of control children (see also [20]). In contrast, Daugherty and Quay [11], and Avila and colleagues [12] did not find a correlation between DRL efficiency and ratings of ADHD symptoms in school-age children. Despite these disagreements, Gordon's procedure is widely used for diagnosing impulsivity in ADHD [21].

Assays of impulsivity based on DRL in the Spontaneously Hypertensive Rat (SHR), an animal model of ADHD, have also been problematic. Bull and colleagues [22] showed that SHR were more efficient than Sprague-Dawley (CD) rats in DRL 60 s, suggesting that SHR were *less *impulsive than a conventional laboratory strain of rats – if indeed inefficiency is a valid measure of impulsivity. Using DRL 72 s, van den Bergh and colleagues [23] concluded that Wistar (WST) rats, another conventional laboratory strain, were not more efficient than SHR. Furthermore, these authors did not observe significant improvements in SHR performance when the rats were administered methylphenidate, a drug shown to enhance inhibitory control of ADHD patients [24,25]. Using a modified version of DRL which controlled for rate of reinforcement, Sagvolden and Berger [26] found that SHR responded more than WKY, but it is unclear whether or not the mean waiting time was substantially shorter for SHR than for WKY.

There are various potential explanations for the failure to detect impulsivity in SHR using DRL. It is possible that SHR do not display the kind of impulsivity that characterizes ADHD, despite analogous performance of SHR and children with ADHD in other behavioral tasks [27-29]. Another possibility is that the DRL task does not elicit ADHD-related impulsivity, as suggested by some reports [11,12]. In this paper we consider two alternative accounts of performance in DRL. Both accounts are consistent with the characterization of SHR as a valid animal model of ADHD, and with evidence that response-withholding tasks like DRL tap into ADHD-related impulsivity.

### Impulsivity and incentive motivation in DRL

Failure to detect abnormal levels of impulsivity in SHR may be related to the sensitivity of DRL performance to incentive motivation [30]: A subject that is not motivated to complete the DRL task may engage in other activities, producing longer IRTs that would be wrongly attributed to self control. With very long target times, such as those used by Bull et al. [22] and van den Bergh et al. [23], it is possible that successful performance is a result of periodic desertion of the task due to flagging motivation, rather than controlled waiting associated with self-control. Failure to detect abnormal impulsivity in SHR using DRL may be due to SHR's reduced motivation for the incentive relative to control strains – more frequent desertion of the waiting task may have been wrongly interpreted as more controlled waiting.

To isolate impulsivity from motivation, we propose to complement measures obtained from DRL with those obtained from a lever-holding task (LHT). In LHT, releasing a lever or button is reinforced only if it had been pressed longer than a specified interval; early releases restart the clock but are not reinforced. Variations of LHT have been used as a timing task in animals [31,32].

In LHT and DRL, reinforcement is contingent on emitting a response (lever or button press in DRL; lever or button release in LHT) after withholding it for a minimum time. Both tasks differ in what subjects are required to do between responses: Waiting activities in LHT are restricted to those that keep the switch depressed, whereas in DRL they are anything other than pressing the switch. Like short IRTs in DRL, short response durations in LHT are deemed impulsive. Supporting this characterization of LHT inefficiency, Baldwin and colleagues [33] demonstrated that methylphenidate reduces the variability of response durations and the frequency of very short durations in children with ADHD.

As the case for DRL, the interpretation of LHT performance is not without confounds. A subject exposed to LHT contingencies may produce shorter "impulsive" response durations because of reduced motivation or poor motor control, not because of an impulsive tendency to release the lever early. Conversely, reduced motivation and poor motor control are unlikely to contribute to shorter IRTs in DRL: Smaller rewards yield longer IRTs in rats and pigeons [30]. Longer "self-controlled" IRTs in DRL may result from low motivation to complete the task. Conversely, reduced motivation is unlikely to result in longer response durations in LHT. Thus, demonstration of impulsivity in *both *LHT and DRL cannot be attributed to motor impairment or enhanced motivation. Some studies have taken advantage of the complementary nature of LHT and DRL to interpret the behavioral effects of drugs in rats [34-37] and to evaluate timing in children [37], as is our strategy here.

### Efficiency and peak deviation

A second hypothesis concerning the failure to detect impulsivity in SHR using DRL derives from the use of DRL "efficiency" as an (inverse) measure of impulsivity. Efficiency is typically inferred from the proportion of responses that exceed the target interval. Efficiency measures are intuitive and descriptive, but do not take full advantage of the data for drawing inferences, as would a truly efficient model of the behavior. A change in efficiency indicates that the distribution of IRTs changed, but it does not tell us precisely how it changed [38]. Four responses spaced just under the target interval followed by a reinforced response are given the same efficiency score (20%) as a burst of 4 closely spaced responses followed by a reinforced response; yet the former performance is more inefficient in most senses of that term. Moreover, despite having the same efficiency score, responses that are spaced almost right would score better in most measures of timing than a burst of responses would. DRL efficiency ignores much of the information contained in performance, some of which may be critical to distinguish levels of impulsivity across strains of rats.

Richards and colleagues [38] suggested an analytic technique that might characterize DRL performance using more information than is provided by efficiency measures. Their *peak deviation analysis *identifies deviations in the IRT distribution from a random process, defined as one in which interval terminations occur randomly with constant probability. A constant-rate random process entails a negative exponential distribution of IRTs. In DRL, very short IRTs ("bursts") and IRTs around the target interval ("peak") are typically much more frequent than expected from a constant-rate random process. Using this analysis, van den Bergh [23] showed that bursts tended to be more frequent and peak IRTs tended to be shorter in SHR than in Wistar (WST); these tendencies, however, did not reach statistical significance.

Peak deviation analysis may reveal differences in DRL performance between SHR and control strains, but it is unclear how these differences should be linked to impulsivity. In the following analysis we develop a plausible behavioral mechanism for both DRL and LHT performance, and test it against data collected from SHR and two other strains. Some of the model parameters are directly related to the ability of rats to inhibit responses, and thus provide a measure of impulsivity. We then evaluate differences in these parameters across strains and reconcile our conclusions with prior data reported on SHR impulsivity.

## Experiment 1: Lever holding task (LHT)

### Method

#### Subjects

Eighteen rats (*Rattus norvegicus*; Charles River), 6 of each of 3 strains (Spontaneously Hypertensive Rat, or SHR, Wistar-Kyoto, or WKY, and Long-Evans, or LE; Charles River Laboratories). WKY was selected as control strain because it is the normotensive genetic progenitor of SHR. LE is also normotensive, but genetic differences between SHR and LE are larger than between SHR and WKY and between SHR and other outbred strains such as Wistar. The use of LE facilitated the identification of candidate behavioral deficits that may have been obscured by behavioral peculiarities of WKY or by similarities with SHR in genetic make-up. Approximate age at the beginning of the experiment was 120 days for SHR, 170 days for WKY, and 130 days for LE. Rats were housed individually in a room with a 12:12-hr light:dark cycle, with dawn at 1800 hr; experiments were conducted only during the dark cycle, the behavioral "day" for this nocturnal species.

The rats' running weights were based on 85% of their expected free-feeding weights, as estimated from a logistic function fitted to the provider's growth curves. According to these curves, initial running weights were 290, 315, and 340 g for SHR, WKY, and LE, respectively. Target weights were already essentially asymptotic for SHR and WKY, but for LE were expected to rise by 50 g over the duration of the experiment.

Each rat was weighed immediately prior to an experimental session. When required, supplementary feeding of 8604 rodent chow (Harlan Teklad, Madison, WI) was given at the end of each day, no fewer than 12 hr before the next experimental session. Supplementary feeding amounts were based 50% on current deviations from running weight, and 50% on a 5-day moving average of the amount historically fed. Thus, for example, if a rat was 4 g below running weight, and it had been fed with a daily average of 2 g of supplementary chow during the last 5 days, 3 g of supplementary chow were provided for that day. This mild regression to the mean amount historically fed dampened vicissitudes in supplementary feeding. Water was always available in the home cages.

All animal handling procedures in this study followed National Institutes for Health guidelines and were approved by the Arizona State University Institutional Animal Care and Use Committee.

#### Apparatus

Experimental sessions were conducted in three MED Associates^® ^modular test chambers (305 mm long, 241 mm wide, and 210 mm high), each enclosed in a sound- and light-attenuating box equipped with a ventilating fan. The floor consisted of thin metal bars above a catch pan. The front and rear walls and the ceiling of the experimental chambers were made of clear plastic, with the front wall hinged and functioning as a door to the chamber. A square aperture (51 mm sides) located 15 mm above the floor and centered on an aluminum side panel provided access to a receptacle (ENV-200-R2M) for 45 mg food pellets (Noyes Precision pellets, Improved Formula A/I, Research Diets, Inc., New Brunswick, NJ). Single pellets were delivered by each activation of a dispenser (ENV-203). Two retractable levers (ENV-112CM) flanked the food hopper. Only the lever closer to the chamber door, to the right of the hopper, was operative; the other lever remained retracted throughout the experiment. The center of the lever was 80 mm from the center of the food hopper, and 21 mm from the floor. Lever presses were recorded when a force of approximately 0.2 N was applied to the end of the lever. A clicker (MED ENV-135M) was located at the top left corner of the test panel; it was used to generate a unique salient cue that indicated food delivery. The ventilation fan mounted on the rear wall of the sound-attenuating chamber provided masking noise of approximately 60 dB. There was no illumination of the test chambers during experimental sessions. Experimental events were arranged via a Med-PC^® ^interface connected to a PC controlled by Med-PC IV^® ^software.

#### Procedure

##### General Design

In the lever-holding task (LHT), holding down and then releasing a response lever was reinforced with food if the time it was held fell within an adjusting criterial range. The lower bound of the range was fixed during experimental sessions and served as target time (*T*), the minimum time rats had to hold for food. The upper bound of the range was adjusted to ensure that about two thirds of response durations longer than *T *were reinforced; the non-reinforced third were the longest durations. Upper-bound adjustment is analogous to the "limited hold" procedure used in DRL, where non-reinforcement of the longest IRTs is intended to discourage task delinquency; it allows for a direct comparison between LHT and DRL (with limited hold) performance. Target times were established in ascending order: 0.25, 0.5, 0.75, 1, 1.5, 2.25, 3.38, and 5 s. The lower bound of the range of reinforced durations was adjusted to transition between target times. Further procedural details are described below.

##### Outline of experimental sessions

Each session started with a 280 s habituation period, during which the response levers were retracted. Each trial was initiated by the insertion of the lever. If the rat held down the lever for more than 100 ms before releasing it, the response duration was recorded and the lever was temporarily retracted upon release. Response durations that fell within an adjusting criterial range were reinforced with two clicks separated by 0.5 s, followed by a food pellet. Durations outside that range were not reinforced. Inter-trial intervals (ITI) of 7.5 s started after reinforcement, or after lever retraction if there was no reinforcement; levers remained retracted during the ITI. Sessions ended after 200 reinforcers or 2 hr, whichever happened first.

##### Adjustment of criterial range

**Figure 1:**
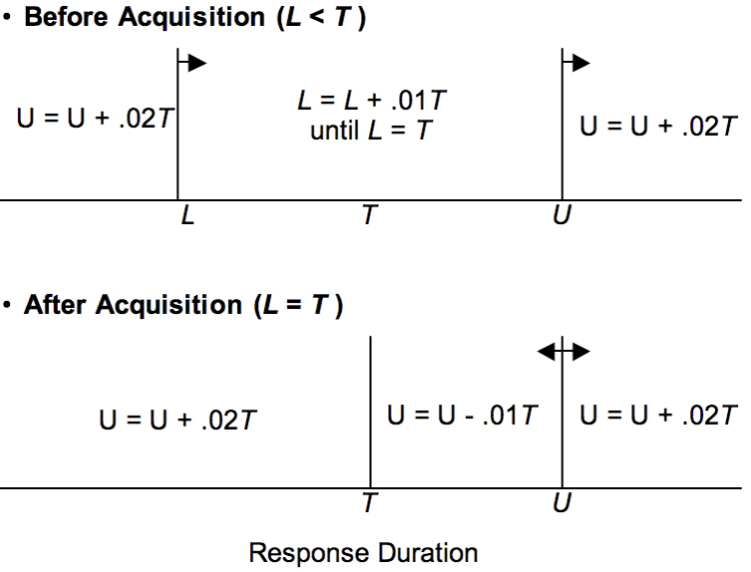
**Range-adjustment procedure in LHT**. Diagram of the procedure for adjusting the range (*L*, *U*) of reinforced response durations. The reinforced range is delimited by vertical lines. The arrows indicate the direction in which the bounds may be adjusted. Equations indicate how the bounds are adjusted as a function of response duration.

The procedure for adjusting the criterial range is diagrammed in Figure 1. At the beginning of the first experimental condition, the lower bound *L *of the criterion was set to zero and the upper bound *U *to 2 s. Response durations anywhere outside the criterion increased *U *by .02*T*, where *T *was the target time (initially set at 0.25 s). Durations within the criterion increased *L *by .01*T*, but not above *T*. After the lower bound reached it, target time *T *was considered *acquired *when 60 pellets were obtained within a session. Following acquisition, *U *was adjusted according to a 2-up-1-down algorithm (reinforced durations reduced *U *by .01*T*; unreinforced durations increased *U *by 0.02*T*).

Once a target time was acquired, at least 2 more sessions (typically 4) were conducted before further increasing the target time. With every new target time, *L *was set to the prior target time; if necessary, *U *was adjusted to ensure that it was not below the new target time. The rules for the acquisition of new target times and for the adjustment of the reinforced range were always as depicted in Figure 1.

##### Inclusion criteria

Rats that had not acquired a target time after 10 sessions were deemed "slow learners" and excluded from further participation in the experiment; data from prior target times, however, was used in analysis. To avoid overrepresentation of "fast learners" in a strain, the exclusion of a slow learner in a strain was compensated by the exclusion of the slowest learner (the one that took longest to acquire the target time that caused the exclusion) in each of the other strains.

Data analysis was based on performance on the last 2 sessions of each target time that was acquired in fewer than 10 sessions. Response durations were deemed stable for a strain during these 2 sessions if the median change between both sessions was less than 6%.

### Results

Rats took between 43 and 55 days to complete Experiment 1 [Additional file 1]. The median rat took 1 session to acquire *T *< 1.5 s and 2 sessions to acquire *T *≥ 1.5 s. Only 3 rats failed to acquire a target time in less than 10 sessions (S4 at *T *= 1.5 s; S6 and W4 at *T *= 5 s). To compensate for their exclusion, data from W2 and L7 were excluded from *T *= 1.5 s and above, and data from L2 were excluded from *T *= 5 s. Rat L3 was accidentally not trained on *T *= 1.5 s, so data for this rat in this condition is missing – no compensation for "slow learning" was conducted. Response durations were stable for all strains in all target times, except for LE rats in *T *= 5 s and WKY rats in *T *= 0.25 s, where the median response duration increased by 12% and decreased by 10%, respectively, over the last 2 sessions.

**Figure 2:**
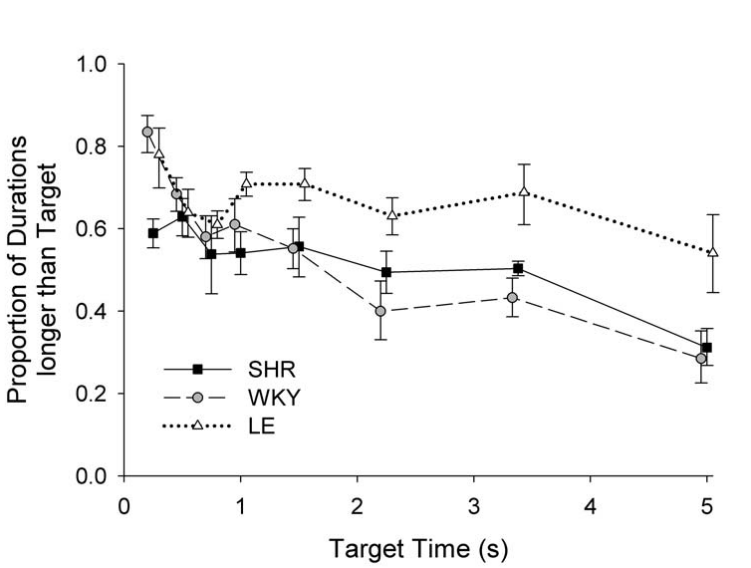
**LHT efficiency**. Mean efficiency in LHT ± SEM as a function of target time *T*. Efficiency was computed as the proportion of durations longer than *T*. For *T *> 1 s, LE rats were substantially more efficient than SHR and WKY rats.

Figure 2 shows the proportion of response durations greater than target time *T*; because this metric indicates the proportion of responses that were reinforced, it has traditionally served as a measure of response efficiency. At *T *= 0.25 s, LE and WKY rats were more likely to produce long durations than SHRs. At *T *= 0.5 s and *T *= 0.75 s, the proportion of long durations in LE and WKY decreased to SHR levels. At longer target times, LE rats produced longer durations than either SHRs or WKY rats. Thus, by traditional inference from differences in efficiency, SHR were more impulsive than WKY for *T *= 1 s, but SHR were more self-controlled than WKY for *T *> 1. Neither of these strains showed anything like the self-control of LE for *T *> 1 s. Whether performance at target times less than one second speaks more to self control or to motor control is moot.

Very long response durations can be even more inefficient than very short ones; a short response just under *T *can at most halve the rate of reinforcement; a long response can reduce it as 1/*t*. A more direct measure of efficiency is rate of reinforcement – the average number of reinforcers obtained on a minute of experimental session while the lever was extended. Applying this metric to LHT 5 s, LE was more efficient than SHR, collecting 5.6 reinforcers per minute, and SHR at 2.2 was more efficient than WKY at 1.6 reinforcers per minute.

**Figure 3:**
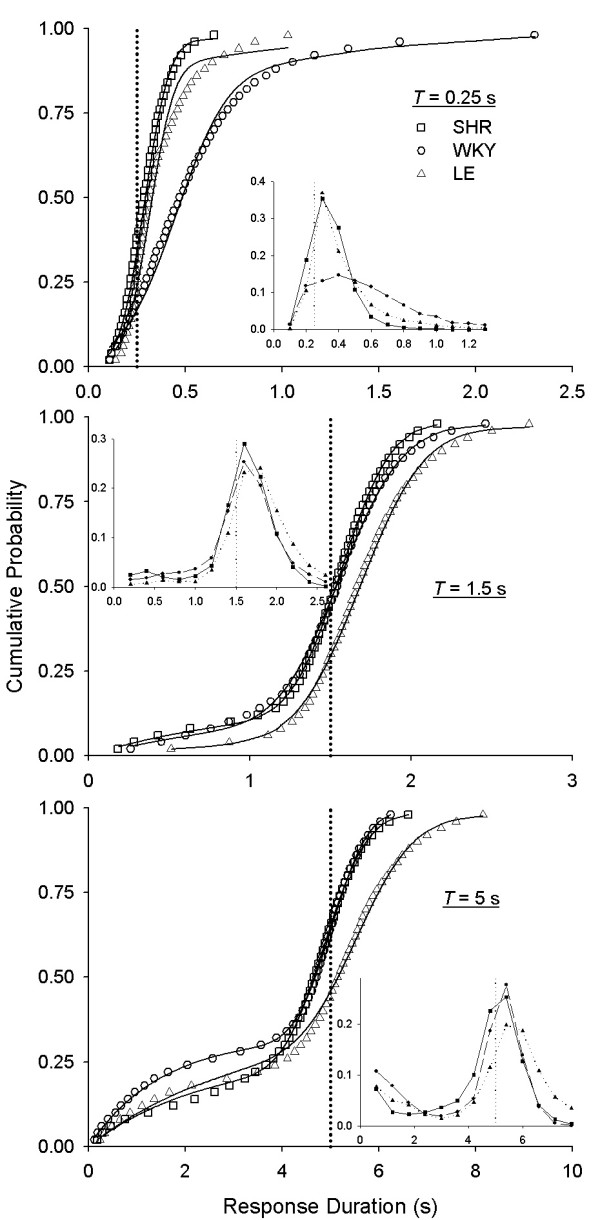
**Pooled LHT performance in a selection of three target times**. Cumulative probability distributions of response durations [*p*(duration <*x*)] for 3 target times (*T*). Distributions were constructed by pooling data across rats of each strain. Insets are corresponding probability densities; strains are identified by corresponding symbols. Curves in the cumulative distributions are fits of the Temporal Regulation (TR) model (Equation 1) to the performance of each strain. The distribution of response duration tracked the target time (vertical dotted lines). At *T *= 5 s, an extra inflexion is visible on the left side of all distributions.

Efficiency is only one index of LHT performance; distributions of response durations provide a more complete picture. To illustrate this, Figure 3 shows the cumulative probability distribution of response durations for 3 target times *T*, pooled within each strain. Whereas probability density functions are more intuitively compelling, they require that data be binned, and they are sensitive to the amount of data in each bin; cumulative distributions are less sensitive to data aggregation procedures. To ground these cumulative distributions to intuitions, the insets in each give the corresponding densities. Each curve in Figure 3 shows the probability *y *that a response duration was shorter than *x*. Efficiency (proportion of reinforced responses) may be directly recovered from these plots as 1 - *B*(*T*) where *B*(*T*) is the ordinate corresponding to an abscissa of *x *= *T*, designated by the vertical dotted lines. Simply extend a horizontal line from the intersection of the dotted line with the curve, over to the *y*-axis; efficiency is the complement of that intercept.

The midpoints and slopes of cumulative distributions in Figure 3 were correlated with *T*, but for longer values of *T *distributions showed an extra inflection on the left side (see the bottom panel of Figure 3). This inflection represents a distinct population of short duration responses. What controls the parameters of this population of response durations? One possibility is that these were carried over from prior target times, but this is unlikely, for three reasons: 1. At short *T*, before the inflection in the distribution appeared, the probability density function of response durations was inverted-U shaped (e.g., Figure 3, inset of top panel), whereas the function of the inflection decreases monotonically as response duration increases. 2. When the inflection appeared, it increased the relative frequency of very short durations. 3. Such bimodal distributions have been reported before under widely different pre-training conditions, both in LHT (e.g., [34]) and for IRTs in DRL (e.g., [38]).

We suggest here that the bimodal distribution of response durations may be the output of two mechanisms operating in temporally regulated responses: A timing mechanism that produces a distribution of durations around the target times, and a random response process that produces shorter durations. The operation of these mechanisms is described more precisely by the Temporal Regulation (TR) model.

#### Temporal Regulation (TR) model

**Figure 4:**
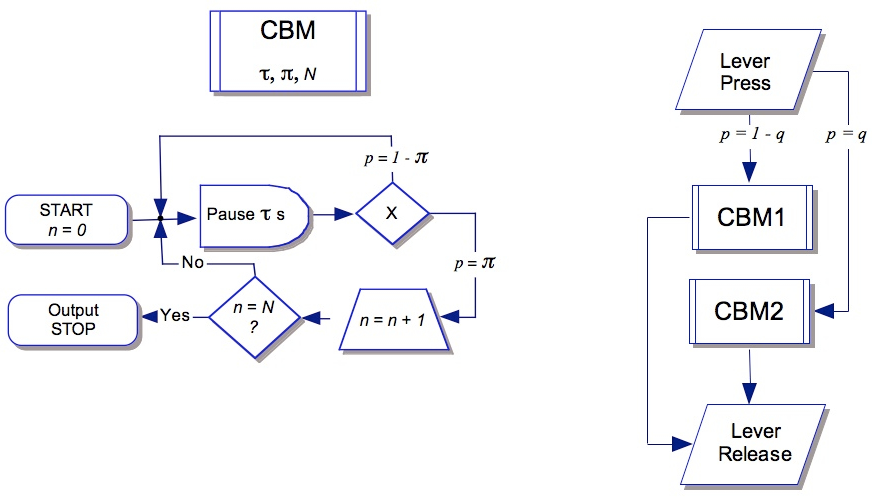
**Temporal Regulation (TR) model represented as a mixture of two CBMs**. Left: A *Clocked Bernoulli Module *(CBM) queries a random probability gate every τ seconds and with probability π then increments a counter. After *N *successes the module outputs and stops. Right: Two CBMs generate the cumulative distribution functions shown in Figure 2. CBM1 is responsible for the iterative responses, which occur with probability 1 - *q*; with complementary probability the animal will move into a timed response for which CBM2 is the mechanism.

TR assumes that a proportion *q *of the response durations in LHT are controlled by the target time; the remainder (1 - *q*) are controlled by a random process similar to flipping a biased coin every so often and responding when it comes up heads. The former we call "timed responses", the latter, "iterative responses." This flow of behavior may be represented as a two step process. A key part of each process is the *clocked Bernoulli module *(CBM) [39]. In this subroutine, a probability device is queried every τ seconds, and with probability π a counter is incremented. As soon as the count *n *exceeds *N*, the process terminates (Figure 4, left diagram).

The familiar process of flipping a coin until it lands heads *N *times constitutes a CBM, with the time between flips equal to τ, and the probability of a head equal to π. A mixture of two CBMs represent the timing mechanism in TR (Figure 4, right diagram). The parameters for these CBMs are given in Table 1. For the iterative module, *N *= 1, resulting in a geometric distribution of response durations with a minimum duration δ. For the timing CBM *N *is larger, generating a Gamma, or Erlang (a Gamma process with integer counts) distribution of response durations. It is possible to further simplify the model, as both CBMs provide more detail than necessary. Although responses cannot be shorter than δ, we may avoid stipulating that parameter by passing from this geometric process to its exponential limit. In like manner, it is unnecessary to identify the minimum dwell in the timing module [40] if we pass to its limiting normal distribution.

**Table 1:** Characteristics of the Temporal Regulation (TR) model

Parameter	CBM1	CBM2
τ	δ	*D*
π	*P*	*P'*
*N*	1	> 1
Cumulative distribution function (cdf)	Geometric	Erlang, Gamma
Limiting cdf	Exponential	Normal
Relative parameters	Mean iterative dwell = 1/λ	θ = μ/T; *w *= σ/μ

If the probability of entering the timing state is *q*, then the distribution resulting from the mixture of these two processes is:

$$B\left(t\right)=q\Phi_{T\theta ,\sigma^{2}}\left(t\right)+\left(1-q\right)\left(1-e^{-\lambda t}\right),$$

where *B*(*t*) is the proportion of response durations shorter than *t*, Φ is the normal cdf (mean μ = *T*θ, variance = σ^2^) of response durations outputted by the timing process, and λ is the iterative response rate. The inferred value of θ = μ/*T *indicates when timed responses are emitted relative to target, and thus serves as a measure of response inhibition in TR that may be compared across different target times *T*. In this pacemaker-counter timing module, the criterial value of *n*, *N *(Figure 4, left diagram), is conditioned by reinforcement, wherein values of criterion *n *associated with *t < T *are extinguished, and larger values (under the upper limit *U*) are reinforced.

#### Data analysis

We focus our analysis on the timing parameters of TR, θ and σ, because these are directly relevant to the evaluation of impulsivity and to the question of its basis in timing deficits. As pointed out by Richards and colleagues [38], the iterative response bursts are highly variable across animals, which obscure a meaningful interpretation of their parameters (λ or τ). Timing parameters were analyzed to determine whether differences across strains were reliable. Parameters were estimated for each rat by using Solver in Microsoft Excel to minimize the sum of squared deviations between the predictions of Equation 1 and the observed distribution of response durations. A protocol for this analysis based on Microsoft Excel is provided in the appendix [Additional file 2].

##### Mean temporal estimates: Accuracy

Changes in the distribution of timed responses as a function of target time *T *have been well described in the timing literature [41,42]. Based on these precedents, we predicted a constant relative response threshold θ across *T*. Because the mean timed response is represented in TR as μ = θ*T *+ δ, θ can be efficiently estimated as the slope of a linear regression of μ on *T*. Responses cannot be of zero duration; the intercept of the regression, δ, estimates the minimum response duration in these circumstances. For large values of *T *the minimum can be set to zero, thus eliminating a nuisance parameter; but for the very small values of *T *studied in this experiment, that is not always possible. A model comparison determined which parameters of the linear regression (θ, δ, both θ and δ, or none) were required to vary across strains in order to describe changes in μ as a function of *T*. The criterion for model selection was determined using the corrected Akaike Information Criterion (AICc) [43]. AICc increases with the residual sums of squares and with the number of free parameters; a smaller AICc thus indicates a better account of the data for the degrees of freedom (parameters) in the model. AICc is only meaningful as a relative value, therefore ΔAICc was computed for each model as the difference between its AICc and the lowest AICc. The most parsimonious model, with ΔAICc = 0, was selected if no other model was at least 10 points better. This process was repeated until all combinations of parameters had been explored. This algorithm guaranteed that the data were at least *e*^10 ^times more likely given the selected model compared to more parsimonious models. Confidence in the model selection was computed using Killeen's *p*_*rep *_[44] for AIC comparisons between nested models [45]. *p*_*rep *_is the estimated probability that the selection would be replicated, that is, that a replication would favor the selected model and not the best simpler model.

##### Variability of temporal estimates: Precision

To assess changes in the precision of the timing process, we describe changes in σ using the Generalized Weber's Law (GWL): $\sigma =\sqrt{{\left(w\mu \right)}^{2}+c^{2}}$, where *w *is the Weber fraction that represents proportional error in temporal discrimination, and *c *is constant error [46]. A model comparison based on AICc, described above, determined which GWL parameters (*w*, *c*, both, or none) were required to vary across strains in order to describe changes in σ as a function of μ.

#### Estimation of TR parameters and model comparison

**Figure 5:**
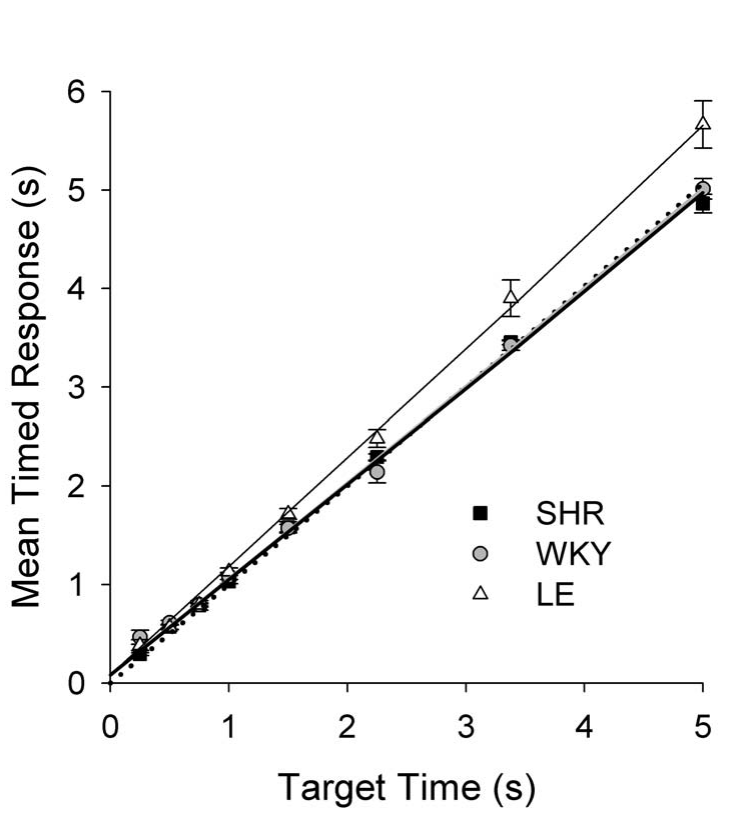
**Timing accuracy in LHT: Mean timed responses**. Mean timed response μ ± SEM as a function of target time *T*. The solid lines are traces of the timed response function μ = θ*T *+ δ, with response threshold θ (but not minimum duration δ) varied across strains. The larger value of θ_LE_, evidenced by the steeper slope of its timed response function, is indicative of a bias towards responding later. For SHR and WKY, the timed response function is closer to the identity (dotted) line, indicating that θ_SHR _≈ θ_WKY _≈ 1.

The proximity of the curves in Figure 3, drawn by Equation 1, to their data show that TR provided a good account of the pooled distribution of response durations. Figure 5 shows that mean timed responses closely followed *T*, represented by the dotted line. The model-comparison analysis (Table 2) supported an account of the data based on different slopes for different strains (θ_SHR _= 0.97, θ_WKY _= 0.97, θ_LE _= 1.10) and a single minimum response duration (δ = 85 ms); *p*_*rep *_> 0.99. Regressions based on these parameters are plotted in Figure 5. Consistent with the greater efficiency of LE shown in Figure 2, SHR and WKY rats produced timed responses that virtually matched *T*, whereas LE rats produced more "conservative" timed responses, 10% longer than *T*.

**Table 2:** Model-comparison analysis for LHT: Timing accuracy and precision

	Parameters varied across strains			
Accuracy	θ and δ	Only θ	Only δ	None
Number of free parameters (*k*)	7	5	5	3
ΔAICc	0.5	0	45.8	65.1

Precision	*w *and *c*	Only *w*	Only *c*	None
Number of free parameters (*k*)	7	5	5	3
ΔAICc	-7.5	0	24.7	23.7

*Note*. AICc for each model was calculated as AICc (model) = 2*k *+ *n *ln(RSS/*n*) + [2*k *(*k *+ 1)/(*n *- *k *- 1)], where *k *is the number of free parameters (3 strains × parameters varied + parameters not varied + 1 variance parameter) and *n *is the total number of observations (18 rats × 8 target times - 16 empty cells = 128). ΔAICc is the difference between AICc of each model and AICc of the selected model; ΔAICc of the selected model is, thus, zero. Some models with lower indices were not selected because they did not improve the account by at least 10 points.

**Figure 6:**
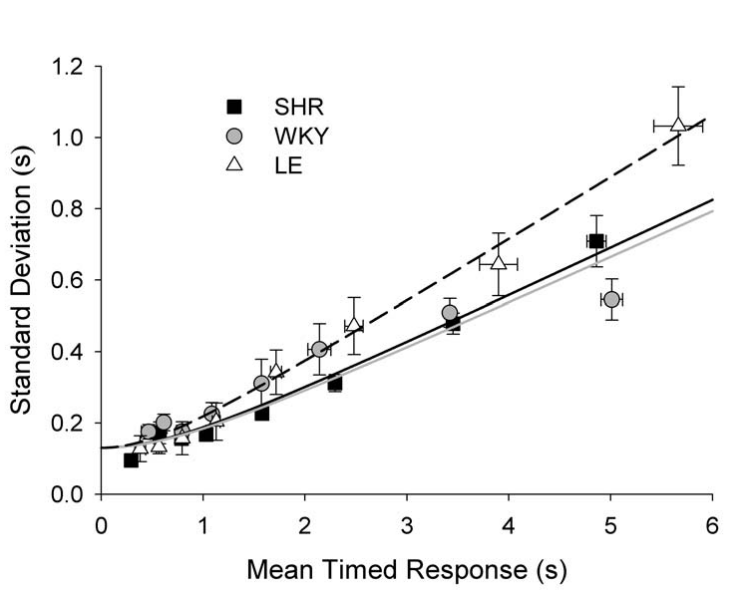
**Timing precision in LHT: Weber functions**. Mean standard deviation of timed responses (σ ± SEM) as a function of mean timed response (μ ± SEM). The solid lines are best fits of the Generalized Weber's Law with Weber fraction *w *(but not constant error *c*) varied across strains. A model-comparison analysis selected the varied parameter. The larger value of *w*_LE _is evidenced by the steeper slope of its Weber function; it is indicative of lower precision in timing.

Figure 6 plots σ against mean timed response. As expected from the literature, σ increased as an approximately linear function of *T*, a property known as scalar timing [41]. Growing out of a constant minimum, the function quickly assumes linearity for SHR and LE, a pattern well described by the Generalized Weber's Law [42]. For WKY, however, the growth of σ flattened at 0.5 above *T *= 3 s, a pattern that is inconsistent with Weber's Law. The curves in Figure 6 are traces of GWL obtained from the model comparison analysis (Table 2). This analysis indicated that the three strains differed in Weber fraction *w *(*w*_SHR _= 0.13, *w*_WKY _= 0.14, *w*_LE _= 0.18) but not in constant error *c *(130 ms); *p*_*rep *_> 0.99. Freeing both *w *and *c *yielded a smaller error variance, but not sufficiently small to justify more free parameters. The large value of *w *for LE corresponds to its steeper Weber function, obvious in Figure 6.

### Discussion

LHT performance by SHR, WKY, and LE rats was well described by the mixture of exponentially and normally distributed response durations of the TR model. For all strains, the sensitivity of the normally distributed portion of the response durations to the target time supports its characterization as the output of a timing process. For SHR and LE it is a simple scalar process, whereas for WKY, the dispersion of the normally distributed durations did not accord with Weber's Law [47-49], increasing faster up to about 2 s and slower thereafter.

Mean timed responses were generally shorter for SHR and WKY than for LE rats, suggesting that SHR and WKY rats have a bias to release the lever earlier than LE rats. This result is consistent with SHR as an animal model of the response inhibition deficit that characterizes ADHD [50], but it is inconsistent with the use of WKY as a control strain: If SHRs are just as impulsive as WKY rats, the source of impulsivity may lie in their common genetic background, thus rendering SHR-WKY comparisons uninformative. The performance of SHR and WKY was similarly suboptimal, resulting in lower efficiency in producing intervals longer than the target time.

The dispersion of timed responses of SHR and LE, but not WKY, was well described by the linear function known as the Generalized Weber's Law. The parameters of SHR suggest that timing precision was not compromised in SHR: if anything, they are more precise than LE. Timing precision in SHR appears to be inconsistent with recent reports of poor time reproduction – the closest analog to LHT – in ADHD [51]. These investigations, however, also indicate that ADHD patients, like SHRs, systematically underestimate long target intervals [52-57]. Only one of these reports describes a measure of precision that allows an estimation of Weber fraction *w *[57]. This research indicates that children with ADHD are not only more impulsive than controls, but that their timing is also less precise. SHRs showed analogous impulsivity relative to LE, as shown by θ_SHR_, but no comparable timing imprecision.

In synthesis, the idiosyncratic performance of WKY in LHT suggests that it may not be the appropriate control strain to evaluate impulsivity in SHR. A comparison between SHR and LE performance suggests that SHRs are less capable of inhibiting reinforced responses – the critical feature of ADHD-related impulsivity. This result may stem from an inhibitory deficit intrinsic to the SHR strain which would support its use as an animal model of ADHD-related impulsivity. This result could also be interpreted as a difficulty in holding down the lever for SHR, relative to LE, due to a motor limitation or its lower body weight; it could also be interpreted as diminished motivation due to lower rates of reinforcement (in turn derived from inefficient responding). Experiment 2 was intended to disambiguate these alternate interpretations of LHT data.

## Experiment 2: Differential reinforcement of low rates (DRL)

Whereas LHT performance may be affected by the physical demands of holding down the lever, this factor is unlikely to be confounded with response inhibition in DRL performance. Moreover, shorter response durations in LHT would result in differences in rate of reinforcement that could further *shorten *response durations in a positive feedback loop; in such case, low rates of reinforcement in DRL would *lengthen *IRTs, which would increase rate of reinforcement, negatively feeding back IRTs. To the extent that rate of reinforcement influences response withholding, LHT and DRL performance should diverge.

To verify our interpretation of LHT data in terms of impulsivity and timing precision, we conducted 3 variations of DRL. First, rats were exposed to a conventional DRL 5 s schedule. Then, we instituted a DRL 5 s schedule with limited hold (DRL-LH 5 s), where very long IRTs were not reinforced. DRL-LH 5 s served a double purpose: First, a comparison between DRL 5 s with and without limited hold assessed the sensitivity of impulsivity and timing to the discouragement of long waiting times. Second, DRL-LH 5 s data was more directly comparable to LHT data, because both schedules had an upper limit. Finally, a DRL 60 s schedule was implemented, which also served two purposes: First, it verified that our conclusions could be generalized beyond *T *= 5 s, and second, it replicated Bull et al.'s [22] study, in which SHRs did not display impulsivity.

## Method

### Subjects and Apparatus

The same 18 rats of Experiment 1 served, after approximately 60 days of resting, under the same housing, feeding, and handling conditions as in Experiment 1. The experiment was conducted in the same chambers used in Experiment 1, but the clicker was replaced with a Sonalert 2.9 kHz tone generator (ENV 223AM).

#### Procedure

##### Differential Reinforcement of Low Rates (DRL) 5 s

After a 300 s habituation period, the lever was inserted. Each lever press that occurred within 100 ms of the prior response was ignored by the program as a lever-bounce. Each valid response started a timer and the next lever press stopped the timer. Inter-response times shorter than 5 s restarted the timer; IRTs longer than 5 s were always followed by a 0.5-s tone simultaneously with the delivery of a food pellet. The first response after a feeding started the timer again. Daily sessions ended after 150 pellets had been delivered or 55 min had elapsed, whichever happened first. Ten sessions were conducted.

##### DRL with Limited Hold (DRL-LH) 5 s

An adjusting limited hold was imposed on the DRL 5 s procedure. IRTs longer than the hold restarted the timer and were not followed by food. The upper bound of the hold was seeded at 10 s; it was adjusted down by 0.01 s after every feeding, and up by 0.03 s after every interval longer than the upper bound. Note that the limited hold in DRL works as the upper limit *U *in LHT, and its adjustment here parallels the adjustment of *U *in Experiment 1 after target acquisition. Thirteen sessions were conducted.

##### DRL 60 s

The DRL target time was increased substantially to replicate the contingencies of Bull et al. [22], who did not find support for impulsivity in SHR. It also approximates the conventional 72-s target time (*T*) used on impulsivity and anti-depressant assays based on DRL [23,58]. Three changes were introduced to the DRL procedure. (a) The timer was restarted by *every *lever press. This meant that after a feeding, the rat did not have to press the lever to start the timer because the timer had been running since the reinforced lever press. (b) The limited hold was removed, and (c) after every feeding, *T*, originally 5 s, was increased by 0.75%. The adjusted *T *was carried over from session to session. Once it reached 60 s,*T *was kept constant at 60 s for 10 sessions.

### Data analysis

Analysis was conducted on IRTs from the last 3 sessions of DRL 5 s and DRL-LH 5 s. Because response rates were substantially lower in DRL 60 s, analysis was conducted on the last 5 sessions of this schedule. IRTs were binned so that each bin contained 1% of a rat's data. Cumulative distributions similar to those of Figure 3 were constructed for each strain. A model-comparison analysis was conducted to determine whether TR's timing parameters were reliably different across strains. Once again, we asked whether θ and *w *varied across strains. Four models were compared using AICc. One model allowed none of the timing parameters to vary across strains; a second model allowed θ but not *w *to vary; a third one allowed *w *but not θ to vary; a fourth one allowed both parameters to vary. When fitting each model, estimates of *q *and λ were fitted to each individual animal. Model selection rules and confidence computation were the same as in Experiment 1. For comparison, this analysis was also conducted on the LHT 5 s data analyzed in Experiment 1.

## Results

**Figure 7:**
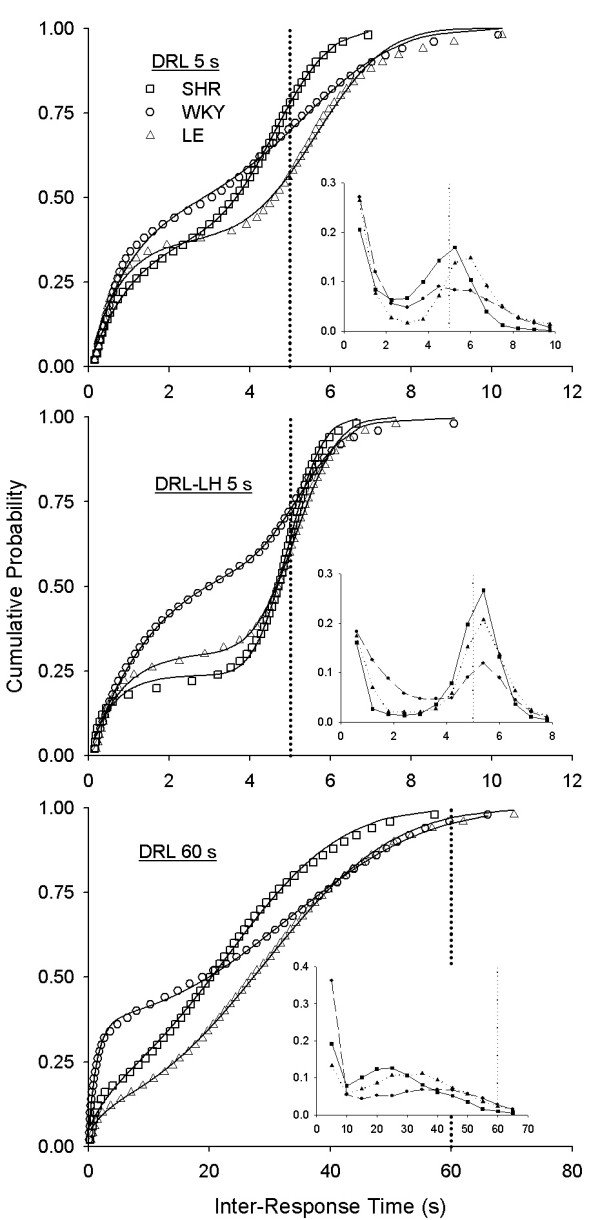
**Pooled DRL performance**. Cumulative probability distributions of IRTs [*p*(IRT <*x*)] in 3 DRL schedules. Distributions were constructed by pooling data across rats of each strain. Insets are corresponding probability densities; strains are identified by corresponding symbols. Curves in the cumulative distributions are fits of the Temporal Regulation (TR) model (Equation 1) to the performance of each strain. The distribution of IRTs was centered near the target time (vertical dotted lines) in DRL 5 s and DRL-LH 5 s, but not in DRL 60 s. Fewer than 4% of IRTs were reinforced in DRL 60 s.

Figure 7 shows cumulative distributions of IRTs pooled within strain and the best fit of Equation 1 for each strain in each DRL schedule. Efficiency (proportion of reinforced responses) may be recovered from these plots as the length of the dotted line above cumulative distributions (1 - *y *when *x *= *T*). In DRL 5 s, LE rats were on the average more efficient than WKY rats, which in turn were more efficient than SHRs. The efficiency of LE rats was markedly reduced when the limited hold was introduced, which yielded a smaller average difference in efficiency between strains. This was because, without limited holds, LE rats were very "conservative," producing long intervals that were not reinforced when the limited hold was instated. Rates of reinforcement were higher for LE (6.5 and 4.5 reinforcers per min, without and with limited hold, respectively) than for SHR (4.1 and 3.8) and WKY (5.3 and 3.7).

The time to transit between DRL-LH 5 s and DRL 60 s varied widely between strains and within the LE strain. The mean number of sessions it took to reach the 60 s target was 51.8 (SD = 8) for SHR, 24.3 (SD = 4.4) for WKY, and 27.7 (SD = 12.1) for LE. As shown in the bottom panel of Figure 7, efficiency of all strains showed a disastrous decrease to less than 4% when the target time was 60 s.

**Figure 8:**
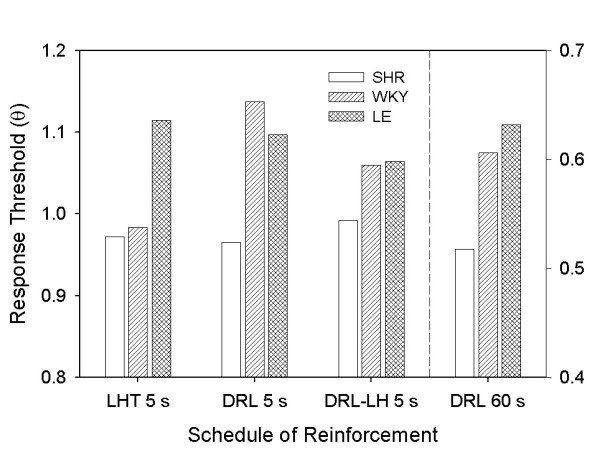
**Response thresholds in LHT 5 s and DRL schedules**. Estimates of response threshold θ based on TR (Equation 1), in LHT and DRL schedules. Note that DRL 60 s estimates are displayed on a different scale (right axis). A model-comparison analysis favored different estimates of θ across strains. θ_SHR _was systematically lower than θ_LE_; θ_WKY _was similar to θ_SHR _in LHT and similar to θ_LE _in DRL.

Table 3 shows the AICc indices obtained from model comparison. Selected models are shown with ΔAICc = 0; *p*_*rep *_> .99 for all selections. Figure 8 shows response thresholds (θ) derived from model-comparison analysis in LHT 5 s (from Experiment 1) and all DRL schedules. θ hovered around 1 in DRL 5 s and DRL-LH 5 s, indicating that μ ≈ *T *for all strains. This was also the case in LHT, as shown by Figures 5 and 8. When the DRL target time increased to 60 s, however, θ decreased across strains by about 40%. Even so, the model-comparison analysis was sensitive to the systematic differences between strains in all DRL procedures. In LHT 5 s, the difference in θ between SHR and WKY was not substantial, but θ was noticeably larger for LE rats. In all DRL schedules, θ for SHR was smaller than for either of the other two strains.

**Table 3:** Model-comparison analysis for LHT 5 s and DRL schedules

	Model			
	θ and *w*	Only θ	Only *w*	None
*k*	43	40	40	37

ΔAICc				
LHT 5 s	0.0	23.7	419.6	646.3
DRL 5 s	-5.5	0.0	32.3	166.7
DRL-LH 5 s	4.4	0.0	161.0	239.3
DRL 60 s	-7.1	0.0	121.8	2157.0

*Note*. See note in Table 3 for computation of AICc and ΔAICc.

**Figure 9:**
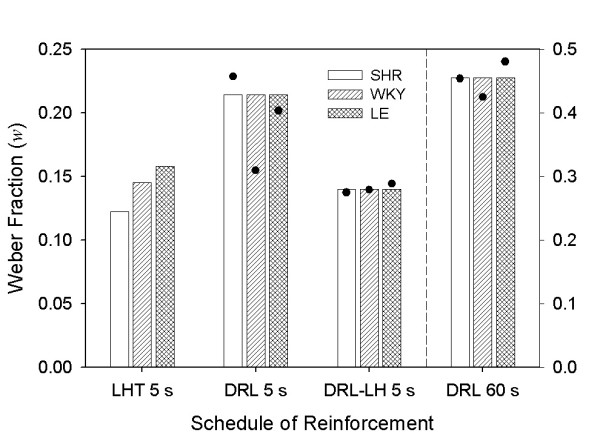
**Weber fractions in LHT 5 s and DRL schedules**. Estimates of Weber fraction *w *based on TR (Equation 1), in LHT and DRL schedules. Note that DRL 60 s estimates are displayed on a different scale (right axis). A model-comparison analysis favored different estimates of *w *across strains in LHT but not in any DRL schedule. The dots indicate the best fitting values of *w *had they been allowed to vary across strains.

Figure 9 shows the estimates of the Weber fraction *w *derived from model comparison analysis in LHT 5 s and all DRL schedules. In the LHT schedule of Experiment 1, SHR were substantially more precise (had lower values of *w*) than LE (see Figure 5). That was not the case for DRL. The performance of all strains was not significantly different from one another according to the AICc criterion (bars, Figure 9) although there was sampling variability across strains (dots). The most noticeable effect was the increase in *w *in DRL 5 and 60 s, when there was no limited hold.

## Discussion

### Response inhibition across tasks and target times

Two features of DRL were varied: the presence or absence of an upper limit (the limited hold) and the target time, 5 or 60 s. In all these variations, the performance of SHR, WKY, and LE rats was well described by the TR model (Figure 7).

One variation in particular, DRL-LH 5 s, is most readily comparable to LHT 5 s, because both schedules have the same target time and an adjusting upper limit. The critical difference between these schedules is what rats were required to do while "waiting": in LHT they had to wait holding down the lever, whereas in DRL they had to wait by staying away from the lever. The complementary nature of LHT and DRL permits establishing whether shorter timed responses are influenced by factors other than impulsivity. Estimates of TR timing parameters from DRL-LH 5 s were close to those obtained from LHT 5 s, suggesting that these parameters are relatively robust over the nature of the waiting responses. Without the limited hold, performance was generally both less accurate (deviations of response threshold θ from 1 were greater in all strains) and less precise (Weber fractions *w *were greater in all strains).

The invariance of parameter values across specific tasks indicates that TR has some generalizability. The relativized timing parameters it provides (θ and *w*) are also invariant across moderately short (1–5 s) target times. However, when the target time of DRL was 60 s, response thresholds declined and Weber fractions increased in all strains. We believe this was due to a mixture of timing responses that usually fell short, and an occasional return from delinquency that was usually reinforced. Lapses of attention may have garnered an occasional reinforcement for flagging performance and resuscitated behavior under DRL 60 s. In LHT, this safety net was not available. These differences are reflected in the fact that it would be virtually impossible for a LHT 60 s schedule to sustain any responding. Our results indicate that different processes may be engaged when target times are very different, which confounds the interpretation of changes in TR parameters across widely different target times.

### Response inhibition across strains

SHRs had lower thresholds for responding (θ) than WKY and LE in DRL schedules, and lower thresholds than LE in LHT schedules. Whereas a reduced ability to press a lever and infrequent engagement in the waiting task would result in shorter LHT timed responses, the same deficits in DRL would result in longer timed responses. The complementary contingencies of LHT and DRL schedules provide converging evidence that impetuous responding reflected in lower values of θ for SHR cannot be attributed to motor deficits or differences in body weight. It is also very unlikely that impulsivity in SHR was confounded with reduced motivation due to low rate of reinforcement; such hypothesis may explain shorter response durations in LHT but not shorter IRTs in DRL. This evidence that response inhibition in SHR is impaired supports the use of SHR as an animal model of ADHD-related impulsivity.

Estimates of θ for WKY were substantially greater on DRL 5 s than on LHT 5 s (but was reined in somewhat by the limited-hold contingency). If WKY were less motivated or less able to work for food than other strains, we would expect increased desertion, which would help DRL performance but not LHT performance. This was just the difference we found. The hypothesis of reduced motivation in WKY is supported by evidence of reduced instrumental responding for sucrose pellets in this strain [59]. Consistent with prior observations [22,60], WKY's different thresholds in the complementary tasks suggests that this strain may not provide consistent baseline levels of response inhibition required for the assessment for impulsivity in SHR.

There were no systematic between-strain differences in the Weber fractions in DRL comparable to those seen in LHT (Figure 5), where LE were less precise than the other two strains. These results confirm that the precision of temporal estimates of SHRs is not compromised, unlike that of children with ADHD [57].

Bull and colleagues [22] compared the distribution of IRTs from SHR, WKY, and Sprague-Dawley (CD) rats under DRL 60 s. Estimates of TR timing parameters from Bull et al.'s data indicate that θ_SHR _= 0.4 and θ_WKY _= 0.69, which are comparable to our estimates (0.52 and 0.61 respectively; see Figure 8). It is surprising, however, that θ_CD _= 0.33, which is even lower than the response threshold of SHR. Compared to our data, CD rats in Bull et al.'s experiment appear to be extraordinarily impulsive. Estimates of *w *in Bull et al.'s rats were substantially larger than in our Experiment 2: Whereas our estimates of *w *in DRL 60 s ranged between 0.4 and 0.5 (Figure 9), estimates for Bull et al's data ranged between 1.2 and 1.6. This is indicative of poorer control of lever pressing by the target time in Bull et al.'s rats. Furthemore, Bull et al's rats were substantially more efficient (10–35%) than ours (1–6%). Several factors in their investigation may have contributed to these effects. Mainly, we suspect that their large target-duration step size used for shaping DRL performance (DRL 30 s immediately preceded DRL 60 s) may have strained waiting times beyond schedule control. In contrast, we introduced DRL 60 s progressively over the course of many sessions, respecting each animal's pace of acquisition. Relative to our data, poorly timed responding in Bull et al's study resulted in flatter IRT distributions and, consequently, enhanced "efficiency". These differences in results underline the importance of shaping procedures in DRL and question the use of efficiency as a measure of impulsivity.

## Conclusion

The failure to inhibit prepotent responses defines the kind of impulsivity that characterizes ADHD. We provided a rationale for using complementary response-withholding tasks to measure impulsivity. In both tasks, the performance of an animal model of ADHD (SHR) and two normotensive strains (WKY and LE) was well described by a mixture of timed and iterated responses; we call this description the Temporal Regulation (TR) model. The TR model delivered a threshold parameter θ, which permitted measurement of bias towards producing short timed responses, independent of the precision of those responses and independent of response bursts. We characterized significant deviations of this parameter below that of control strains as a measure of impulsivity. Lower response thresholds in SHR indicated that they are systematically more impulsive than WKY and LE in a manner that is consistent with the performance of humans with ADHD. This result supports the use of SHR as an animal model of ADHD-related impulsivity. The inconsistent performance of WKY across response-withholding tasks undermines its conventional use as non-impulsive control strain; conventional laboratory strains like LE, Wistar (WST), or Sprague-Dawley (CD) should be considered.

Although SHRs are impulsive, the precision of their temporal estimates is not compromised as has been suggested for ADHD [51]. More research on timing in ADHD is required to verify, across tasks and motivational conditions [61,62], whether or not timing is systematically altered in ADHD. In both animal and human research, we suggest that detailed distributions of response durations and IRTs be reported, so that parameters from TR and from other models of timing and impulsivity may be estimated.

## Competing interests

The author(s) declare that they have no competing interests.

## Authors' contributions

FS collected and analyzed the data and wrote the ms; PRK advised and consented. All authors read and approved the final manuscript.

## Supplementary Material

Additional file 1 Total number of sessions and number of sessions after acquisition. Click here for file

Additional file 2 **Appendix: Model-Comparison Protocols**. Details Microsoft Excel protocols for model-comparison analysis (Microsoft word document: Appendix.doc) Click here for file
