# Supplementary material for: Evidence for impulsivity in the Spontaneously Hypertensive Rat drawn from complementary response-withholding tasks
Source: Behav Brain Funct. 2008 Feb 8;4:7. doi: 10.1186/1744-9081-4-7 (PMC2276225; doi:10.1186/1744-9081-4-7)
Supplement: Additional file 1 — Total number of sessions and number of sessions after acquisition. [file 1744-9081-4-7-S1.doc]

# Appendix: Model-Comparison Protocols

The following protocols detail the procedures conducted for the model-comparison analyses described in this investigation. Each protocol was conducted on a separate Microsoft Excel spreadsheet.

## Temporal Regulation (TR) parameter estimation

For each rat in each target time:

- Arrange response durations vertically in Column A and sort them in ascending order.
- Set the cumulative probability of a response duration in Column B, using the formula =COUNTA($A$1:A*r*)/COUNTA(A:A), where *r* is the corresponding row number.
- Set the logit transformation of cumulative probability in Column C, using the formula =LN(B*r*/(1-B*r*)).
- Set the predicted cumulative probability in Column D, using the formula =*p**NORMDIST(A*r*,μ,σ,TRUE)+(1-*p*)*(1-EXP(-λ*C2)). The symbols are the TR parameters described in the text.
- Set the logit transformation of predictions in Column E, using the formula =LN(D*r*/(1-D*r*)).
- Select an empty cell to be used as “error cell.” The error cell will contain the sum of the square difference between Columns C and E; use the formula =SUMXMY2(C1:C*r_max_*,E1:E*r_max_*), where *r_max_* is the number of responses.
- Run Solver. Select the error cell as target; minimize it by changing the cells containing the parameters.

## Accuracy of temporal estimates

For each model:

- In a 18 row × 8 column space, arrange the estimates of μ for each rat (row) in each target time *T* (column). The top 3 rows contain SHR data, the middle 3 rows WKY data, and the bottom 3 rows LE data.
- In another 18 row × 8 column space, set the predictions of μ using the formula =*T**θ_•_+*m*_•_. θ_•_ and *m*_•_ are the accuracy parameters described in the text. The subscript placeholder (_•_) is SHR in the top 3 rows, WKY in the middle 3 rows, and LE in the bottom 3 rows.
- Select an empty cell to be used as “error cell.” The error cell will contain the sum of the square difference between the two 18 × 8 spaces. The formula should look something like =SUMXMY2(A1:H18,A20:H37).
- In a 2 row × 3 column space, set the accuracy parameters (rows) for each strain (columns). Select the “changing cells” from within this space. Cells in left column are always changing cells. If the model calls for keeping θ constant across strains, set the middle and right cells in the θ row equal to the left cell in the same row (θ_SHR_ = θ_WKY_ = θ_LE_). If the model calls for varying θ across strains, set all the cells in the θ row as changing cells. Repeat this procedure in the *m* row.
- Run Solver. Select the error cell as target; minimize it using the changing cells in the 2 × 3 parameter space.
- Compute AICc using the fomula =2**k*+*n**LN(*RSS*/*n*)+(2**k**(*k*+1))/(*n*-*k*-1), where *k* is the number of free parameters (number of changing cells + 1 variance parameter), *n* is the number of observations (18 rats × 8 target times – 16 empty cells = 128), and RSS is the residual sum of squares (value in error cell).

## Precision of temporal estimates

For each model, follow the steps described for the accuracy of temporal estimates with the following changes:

- Add another 18 × 8 space, which contains the estimates of σ for each rat (row) in each target time *T* (column).
- In the 18 × 8 prediction space, set the predictions of σ using the formula =IF(μ="","",SQRT((*w*_•_*μ)^2+*c*_•_^2)). *w*_•_ and *c*_•_ are the precision (GWL) parameters described in the text. The subscript placeholder (_•_) is SHR in the top 3 rows, WKY in the middle 3 rows, and LE in the bottom 3 rows.
- The error cell contains the sum of the square difference between the 18 × 8 σ-estimates space and the 18 × 8 prediction space.
- In the 2 × 3 parameter space, set the precision parameters (rows) for each strain (columns).
