# Supplementary material for: Evidence for impulsivity in the Spontaneously Hypertensive Rat drawn from complementary response-withholding tasks
Source: Behav Brain Funct. 2008 Feb 8;4:7. doi: 10.1186/1744-9081-4-7 (PMC2276225; doi:10.1186/1744-9081-4-7)
Supplement: Additional file 2 — Appendix: Model-Comparison Protocols. Details Microsoft Excel protocols for model-comparison analysis (Microsoft word document: Appendix.doc) [file 1744-9081-4-7-S2.doc]

Total number of sessions / number of sessions after acquisition

| Rat | Target time (s) | | | | | | | |
| --- | --- | --- | --- | --- | --- | --- | --- | --- |
|  | 0.25 | 0.5 | 0.75 | 1 | 1.5 | 2.25 | 3.38 | 5 |
| W1 | 6/5 | 6/6 | 5/4 | 6/6 | 5/4 | 5/3 | 5/3 | 5/3 |
| W2 | 6/5 | 6/6 | 5/4 | 6/6 | 5/3* | 5/3* | 7/5* | 7/5* |
| W3 | 6/5 | 5/4 | 5/4 | 5/4 | 5/4 | 5/3 | 5/3 | 11/7 |
| W4 | 6/5 | 6/6 | 5/4 | 6/6 | 5/3 | 5/4 | 5/3 | 11/0* |
| W5 | 6/5 | 6/6 | 5/4 | 6/6 | 5/4 | 5/3 | 6/2 | 7/5 |
| W6 | 6/5 | 5/4 | 5/4 | 5/4 | 6/5 | 5/3 | 5/2 | 9/7 |
| S1 | 10/9 | 5/4 | 5/3 | 5/4 | 5/4 | 6/4 | 6/4 | 6/4 |
| S2 | 10/9 | 5/4 | 5/4 | 5/4 | 5/3 | 6/4 | 6/3 | 6/3 |
| S3 | 10/9 | 5/4 | 5/4 | 5/4 | 5/4 | 9/4 | 5/3 | 10/2 |
| S4 | 10/9 | 5/4 | 5/3 | 5/4 | 15/4* | - | - | - |
| S5 | 10/9 | 5/3 | 5/4 | 5/4 | 5/3 | 7/6 | 6/2 | 6/3 |
| S6 | 10/9 | 5/4 | 5/4 | 5/3 | 5/2 | 8/6 | 6/4 | 11/0* |
| L2 | 11/10 | 6/5 | 10/9 | 5/4 | 5/4 | 5/3 | 5/3 | 6/2* |
| L3 | 11/10 | 6/5 | 10/9 | 5/3 | - | 5/4 | 5/3 | 6/4 |
| L4 | 11/10 | 5/4 | 10/9 | 5/4 | 5/4 | 5/4 | 5/3 | 5/3 |
| L5 | 11/10 | 5/3 | 5/4 | 5/4 | 5/4 | 8/7 | 5/3 | 8/6 |
| L6 | 11/10 | 5/4 | 5/4 | 5/4 | 5/4 | 8/7 | 5/4 | 8/6 |
| L7 | 11/10 | 5/4 | 5/4 | 5/4 | 5/4* | 8/7* | 5/3* | 6/4* |

*Data excluded from analysis. See text for details.
